# Genomic insights into genetic diversity and seed coat color change in common bean composite populations

**DOI:** 10.3389/fpls.2024.1523745

**Published:** 2025-01-24

**Authors:** Eva Plestenjak, Mohamed Neji, Lovro Sinkovič, Vladimir Meglič, Barbara Pipan

**Affiliations:** ^1^ Crop Science Department, Agricultural Institute of Slovenia, Ljubljana, Slovenia; ^2^ Biotechnical Faculty, University of Ljubljana, Ljubljana, Slovenia

**Keywords:** *Phaseolus vulgaris*, seed coat color, phenotypic variation, composite populations, whole genome sequencing

## Abstract

**Introduction:**

The color of the seed coat of common bean (*Phaseolus vulgaris* L.) is an important trait influencing marketability and consumer preferences. An understanding of the genetic mechanisms underlying seed coat color variation can aid in breeding programs aimed at improving esthetic and agronomic traits. This study investigates the genetic diversity and molecular mechanisms associated with seed coat color change in composite bean populations through phenotypic analysis and whole genome sequencing (WGS).

**Methods:**

Four composite populations and two standard varieties of common bean were cultivated over a two-year period and seed coat color and morphological traits were assessed. WGS was performed on 19 phenotypes and yielded 427 GB of data with an average sequencing depth of 30×. More than 8.6 million high-confidence single nucleotide polymorphisms (SNPs) were identified. Genetic diversity metrics such as nucleotide diversity (π), observed heterozygosity (Ho), expected heterozygosity (He) and allelic richness (Ar) were calculated. Population structure was analyzed using Fst, principal component analysis (PCA) and clustering. Cross-population statistics (XP-CLR and XP-EHH) were used to identify selection signals associated with seed coat color change. Gene Ontology (GO) and KEGG enrichment analyzes were performed for candidate genomic regions.

**Results:**

Phenotypic analysis revealed significant differences in seed coat color among the four composite populations, with notable changes among years. The populations exhibited different growth habits and plant types, especially KIS_Amand and SRGB_00366, which showed the highest phenotypic diversity in seed coat color. WGS identified 8.6 million SNPs, with chromosomes 4 and 1 having the highest SNP density (11% each), while chromosomes 3 and 6 had the lowest. KIS_Amand had the highest genetic diversity (π = 0.222, Ar = 1.380) and SRGB_00189 the lowest (π = 0.067, Ar = 1.327). SRGB_00366 showed moderate genetic diversity (π = 0.173, Ar = 1.338) and INCBN_03048 showed medium diversity (π = 0.124, Ar = 1.047). The Fst values indicated a strong genetic differentiation, especially between the two standard varieties ETNA and Golden_Gate (Fst = 0.704) and the composite populations. Selective sweep analysis with XP-CLR and XP-EHH identified 118 significant regions associated with seed coat color change, with most regions located on chromosomes 4, 9, 10 and 11. Phosphatidylinositol signaling pathways were highly enriched in candidate regions, indicating that cellular transport mechanisms play a critical role in seed coat pigmentation. Key GO terms included phosphatidylinositol-biphosphate binding, exocytosis, and vesicle-mediated transport, suggesting a link between cellular transport and pigment deposition in the seed coat.

**Discussion:**

The study demonstrates significant genetic diversity within and among common bean composite populations, with KIS_Amand and SRGB_00366 exhibiting the highest phenotypic and genetic variability. The identification of selective sweeps and the enrichment of phosphatidylinositol-related pathways provide new insights into the molecular mechanisms controlling seed coat color variation. The strong genetic differentiation between standard varieties and composite populations highlights the role of selective breeding in shaping the genetic landscape of common bean. The results suggest that variation in seed coat color is controlled by both regulatory and structural genetic changes, providing valuable information for breeding programs.

**Conclusion:**

This study provides a detailed analysis of the genetic architecture of seed coat color variation in common bean. The identification of key genomic regions and pathways associated with seed pigmentation improves our understanding of the complex genetic interactions underlying this trait. These results provide valuable genomic resources for future breeding efforts aimed at improving seed color and other important traits in common bean.

## 1 Introduction

The common bean (*Phaseolus vulgaris* L.) is a widely cultivated legume that serves as a significant source of protein and other essential nutrients for human consumption. These include dietary fiber, complex carbohydrates, minerals, vitamins and phenolic compounds (Bitocchi et al., 2017a; Ganesan and Xu, 2017; Carbas et al., 2020). It is a self-pollinating annual plant with a relatively small diploid genome of 587 Mbp, cultivated primarily for its mature seeds and also for its immature (green) pods, which are utilized as a vegetable (Schmutz et al., 2014; Ntatsi et al., 2018). Following the process of domestication, which led to a reduction in genetic diversity and gene expression, the global dissemination of the common bean in the 16^th^ century gave rise to further changes associated with adaptations to different environmental conditions and cultivation practices (Bitocchi et al., 2017a; Cortinovis et al., 2020; Nadeem et al., 2021). The common bean is currently cultivated in many regions of the world, including Europe, where it plays an important role in countries such as Spain, Italy and France (CBI, Ministry of Foreign Affairs, 2022). There is considerable variation in the preferences for certain bean varieties between countries and even within regions. The continuous process of breeding and developing new varieties is driven by the necessity to enhance plant productivity, quality and resistance to pests and environmental stressors. Moreover, the newly developed varieties must meet the needs of producers and consumers (Assefa et al., 2019). Each new variety must be distinct, uniform and stable (DUS), maintaining the genetic and morphological characteristics that are unique to it. Moreover, the characteristics of the new variety, including yield, resistance, and other quality traits, must be superior to those of existing varieties (Council of the European Union, 1994, 2002; UPOV, 2002; Yang et al., 2021). One of the most significant characteristics of dry common beans for consumers is the color of the seed coat, which can vary considerably. The seed coat can be uniform in color, covering the entire seed (primary seed coat color) or it may have a secondary seed coat color, also known as a pattern, which overlays a light tan background and produces stripes or mottling patterns. Furthermore, seeds may exhibit partial coloration, wherein discrete white and colored zones manifest in various shapes across the seed coat (Bassett, 2007; Lioi and Piergiovanni, 2013; García-Fernández et al., 2021).

The observed seed coat color in common bean is the result of the accumulation of specific compounds in seed coats, namely flavonols (usually colorless to light yellow pigments), anthocyanins (variation of red, purple and black pigments) and proanthocyanidins (colorless pigments that turn brown after post-harvest darkening) (Freixas Coutin et al., 2017). The flavonoid biosynthetic pathway is organized into two distinct categories of genes: structural genes, which encode enzymes involved in different steps of the pathway; and regulatory genes, which govern the activity of structural genes by influencing their transcription process. Three families of transcription factors are involved in the regulation of structural genes: MYB, bHLH (basic helix-loop-helix) and WDR proteins. In dicotyledonous plants, the early biosynthetic genes (EBG) are regulated by MYB, while the late biosynthetic genes (LBG) are regulated by all three families of transcription factors, resulting in the formation of MBW (MYB, bHLH, WD40) complexes. MYB transcription factors play a crucial role in DNA binding and subsequent activation of DNA transcription. The bHLH transcription factor facilitates precise tuning of gene expression and the third component of the MBW complex the WD40 protein, stabilizes the MYB-bHLH interaction and provides structural support for the complex (Freixas Coutin et al., 2017; Paauw et al., 2019; García-Fernández et al., 2021).

The main regulator for the gene expression of seed coat color and flower color is the *P* gene. In plants with a homozygous recessive form of the *P* gene (*pp*), all seeds and flowers are white, irrespective of the presence of other genes responsible for the pigmentation of the seed coat. The protein is a member of clade B of subclass IIIf of plant bHLH proteins (Bassett, 2007; McClean et al., 2018). The influence of additional genes on seed coat color can be classified into two categories. The first group of genes, including *Bic*, *C* (which encodes a MYB transcription factor, *PvMYB113*), *J* (which is suspected to act as a MYB transcription factor), *Prp* ^i^ *-2*, *R-2* and *Sal*, determines the color of the seed coat. The second group of genes, including *B*, *G*, *Rk* (possibly a MYB transcription factor) and *V* (encoding flavonoid 3´5´hydroxylase), influences the intensity of the seed coat color. The primary color, which encompasses the entire surface of the seed coat and can be observed as black, yellow, red, etc., is determined by genes for both seed coat color and color intensity (Bassett, 2007; McClean et al., 2018; Erfatpour and Pauls, 2020; García-Fernández et al., 2021; Celebioglu et al., 2023; Parker et al., 2024). However, numerous genes possess multifunctional characteristics. For instance, locus *C* comprises a set of closely linked genes, including *Prp*, *Prp* ^i^, and *Gy*, which influence on seed coat color. Additionally, *M*, *Pr*, *Acc* and *R*, have been demonstrated to exert an influence on seed coat pattern. Patterns observed include a lighter background color and a darker pattern color, which are classified as pinto, striped and mottled types (Bassett, 2007; Wu et al., 2019). The dominant form of the *T* gene is responsible for the production of fully colored seeds and flowers, whereas the homozygous recessive form (*tt*) results in partial coloration. The gene encodes a transcription factor, WD40. Different mutations and deletions may result in the loss of its function, leading to the different partial coloration of the seeds and it can also influence flowers. The distribution of the colored part is also influenced by the action of other genes, including *Bip* (identified as the PvMYC1 transcription factor), *Fib*, *J* and *Z* (which encodes the MYB transcription factor, MYB113) (Bassett, 2007; Wu et al., 2019; García-Fernández et al., 2021; Parker et al., 2024). A considerable number of these genes possess multiple alleles, exert influence on both primary and secondary seed coat coloration, as well as on the coloration of different plant parts (pod, flower, stem), and exhibit epistatic interactions that result in a wide variety of seed coat colors (Bassett, 2007; McClean et al., 2018, 2022; García-Fernández et al., 2021).

The results of recent studies have provided greater insight into the specific functions of certain genes, however, a significant number of genes remain without a clearly defined function. Despite this progress, the relationship between seed coat color variation and underlying genomic factors remains poorly understood, especially in composite populations where phenotypic diversity is high. In some cases, seeds from the same plant may exhibit variation in seed coat color as a consequence of adaptation to unfavorable environmental conditions, increased outcrossing, mutation or genetic drift (Tiranti and Negri, 2007; Klaedtke et al., 2017; Šajgalík et al., 2019). An understanding of the genetic control of seed coat color variation and how it correlates with population structure could provide critical insights into the adaptive potential of common bean populations. This study evaluated composite populations of common bean, which exhibited differences in primary and/or secondary seed coat color. Each composite population exhibited between two and five distinct seed phenotypes, which were evaluated individually. By focusing on composite populations, this study offers a unique perspective on the genetic diversity within populations that are subject to evolutionary forces such as selection, gene flow and genetic drift. The objective of this research was to examine the genetic diversity and population structure of composite common bean populations, with a particular focus on identifying genetic variations and specific genomic regions associated with seed coat color variation. A comprehensive understanding of the genetic diversity and structure of these populations is essential for elucidating the principles of adaptive evolution and for developing effective breeding strategies. Additionally, our findings provide a foundation for future studies aiming to explore the ecological and evolutionary significance of seed coat color in *Phaseolus vulgaris*.

## 2 Materials and methods

### 2.1 Plant material

A total of 50 composite populations of common bean were obtained from various European research projects, the Slovenian Plant Gene Bank, (SRGB) and the breeding program of the Agricultural Institute of Slovenia (KIS). Individual composite population exhibited between two and five distinct seed phenotypes, which differed in seed coat color (primary and/or secondary) and/or the distribution of secondary color on the seed coat. In total, the common bean composite populations comprise 132 individual phenotypes differing in pattern and/or seed coat color, including two standard varieties (ETNA, Golden_Gate). Based on the highest level of segregation observed after the first year of field evaluation, a subset of four common bean composite populations out of 50 and two standard varieties was selected for this study (**Table 1**). The subgroup thus comprises 19 individual phenotypes, including two standard varieties.

**Table 1:** Subgroup of four composite populations and two standard varieties used in this study, their seed coat color variation, growth habit and plant type.

CPOP/VAR	Sowing 2022		Sowing 2023		Plant type	Growth habit	Source
	Phenotype ID	Seed coat color	Phenotype ID	Seed coat color			
KIS_Amand	19D	black	19D	black	Determinate bush	Determinate	Breeding program at KIS
	20D	grey with black pattern	20D-A	black			
	*	*	20D-B	grey with black pattern			
	21D	black	21D-A	black			
	*	*	21D-B	brown			
	*	*	21D-C	brown with veins and no shine	Indeterminate bush, with erect stems		
	22D	brown	22D	brown	Determinate bush		
SRGB_00189	141	beige with red pattern	141-A	beige with red pattern	Determinate bush	Determinate	SRGB
	*	*	141-B	red with beige pattern			
	142	red with beige pattern	142-A	beige with red pattern			
	*	*	142-B	red with beige pattern			
SRGB_00366	152	brown with green pattern	152-A	beige with green pattern	Indeterminate prostrate, with many lateral guides	Indeterminate	
	*	*	152-B	green with beige pattern			
	153	green with beige pattern	153	beige with green pattern			
	154	beige with black pattern	154	beige with black pattern			
INCBN_03048	94	brown	94	brown	Indeterminate climber	Indeterminate	EU projects
	95	white	95	white			
ETNA	302D	beige with red pattern	302D	beige with red pattern	Determinate bush	Determinate	Standard variety
Golden_Gate	303D	white	303D	white	Indeterminate climber	Indeterminate	

*seed phenotypes did not exist in 2022, new phenotypes emerged in 2023; CPOP, composite population; VAR, standard variety; KIS, Agricultural Institute of Slovenia; SRGB, Slovenian Plant Gene Bank.

### 2.2 Experimental design

A field trial was conducted in two consecutive years, 2022 and 2023, at the Experimental station of the Agricultural Institute of Slovenia in Jablje, Slovenia (302 m a.s.l.; 46.08°N 14.33°E). As these populations are quite rare, the number of seeds available for sowing was limited to a maximum of eight per individual phenotype within a composite population. Prior to sowing, each individual phenotype was photographed, and the seeds were evaluated using 16 morphological descriptors. Sowing took place in mid-May; the individual phenotypes were sown in 1 × 1.2 m plots. Data on the type of growth was also collected before sowing. For the plants with indeterminate growth habit (growing upright by themselves), a support was provided in the form of poles around which the seeds were sown. For the plants with determinate growth habit (those that do not climb and grow as bushes), eight seeds were sown in two rows with a row spacing of 40 cm and a plant spacing of 10 cm in the row. The plants were evaluated during the growing season, including plant morphology, such as plant height, stem diameter, growth habit and plant type. With regard to the latter, the apical bud was observed at the stage of maximum flowering, and accordingly, the plants were grouped into four subgroups. Plants with a determinate growth habit and a bushy type of growth were classified as determinate bushes, while those that produce longer stems were classified as indeterminate bushes with erect stems. Plants with an indeterminate growth habit that reach the middle of poles were classified as indeterminate prostrate with many lateral guides. Those that reach the end of the poles were classified as indeterminate climbers. Based on the morphological data obtained after the first year of evaluation and the highest level of seed coat color segregation, four composite populations were selected for sequencing.

### 2.3 Molecular analysis

DNA was isolated from young trifoliate leaves at Eurofins Genomics (Constance, Germany), where its integrity and quality were thoroughly assessed using their advanced protocols. Following isolation, DNA libraries were prepared, quality checked, pooled, and sequenced by Eurofins Genomics (). Whole Genome Sequencing (WGS) was performed on the Illumina NovaSeq 6000 platform in paired-end mode, with 2 × 150 bp read lengths.

#### 2.3.1 Quality control, variant calling and annotation

After sequencing, paired-end fastq files containing R1 (forward) and R2 (reverse) reads were generated for each sample. The quality of the raw sequence reads was assessed using fastQC v0.12.1 software (Hadfield and Eldridge, 2014). Subsequently, the sequence reads were aligned to the *Phaseolus vulgaris* reference genome assembly of Andean common bean G19833 (PhaVulg1_0) using Burrows-Wheeler Aligner (BWA-0.7.17) software (Li and Durbin, 2010), resulting in Sequence Alignment Map (SAM) files samtools (version 1.3.1) software (Li et al., 2009) was then used to convert mapping results into the Binary Alignment Map BAM format and filter the unmapped reads. The BAM files were sorted using samtools, and variant calling was performed using bcftools-1.17. The read sequences of all samples were merged into a single binary BCF (variant call format) file using the ‘samtools mpileup’ flag. Additionally, the vcftools software (Danecek et al., 2011) was utilized to convert the BCF file to VCF format, and the resulting files were further filtered for downstream analysis using the VcfFilter tool. Only SNPs with high-quality scores (Phred Quality Score Q = 30) and a read depth (RD) of 10 were considered for subsequent analysis. Finally, the retained SNPs were annotated and classified using SnpEff software (Cingolani et al., 2012), based on their impact (high, moderate, modifying, and low), their functional class (synonymous and non-synonymous substitutions), and their genomic regions such as downstream, upstream, exon, intron, intragenic and intergenic regions, transcript and 3′ and 5′ untranslated regions (UTRs). DNA substitution mutations (transitions and transversions) and amino acid changes were also identified.

#### 2.3.2 Genomic diversity metrics and genetic differentiation

The genetic diversity within each population or variety was assessed by calculating general metrics such as observed heterozygosity (Ho), expected heterozygosity (He), nucleotide diversity (π), and inbreeding coefficient (Fis). These metrics were estimated using the STACKS software (Catchen et al., 2011). Additionally, allelic richness (Ar), defined as the average number of alleles per locus across all loci, was estimated using the R package ‘hierfstat’, version 0.5-7 (Goudet and Jombart, 2015). Inbreeding within populations was also assessed using runs of homozygosity (ROH), which were identified using PLINK with the *–homozyg* option and a window of 50 SNPs *(–homozyg-window-snp 50*). Further analyses, including distance-based relationships, principal component analysis (PCA), and genetic clustering, were performed to investigate genetic differentiation and population structure. Genetic differentiation among populations was estimated using the Fst coefficient calculated with STACKS ‘populations’ function v2.60 (Catchen et al., 2013). PCA was performed using the function ‘glPca’ from the R package ‘adegenet’ v2.1.10 (Jombart, 2008; Jombart and Ahmed, 2011), where alleles were treated as units and scaled according to their number. For clustering analysis, the ‘bitwise.dist’ function of the ‘poppr’ package v2.9.4 (Kamvar et al., 2014) was used to calculate the genetic distances between the 19 common bean accessions based on the proportion of loci that differ from each other. A dendrogram was created using the Unweighted Pair Group Method with Arithmetic Mean (UPGMA) algorithm with 1000 bootstrap iterations for branch support, implemented using the ‘aboot’ function from the same package, and visualized using the R package ‘ggtree’ v3.10.0 (Yu et al., 2017).

#### 2.3.3 Selection signals and gene set enrichment analysis

To identify selective regions associated with seed color change in the studied composite populations/varieties, the cross-population composite likelihood ratio test (XP-CLR) and the cross-population extended haplotype homozygosity test (XP-EHH) tests were performed with 50 kb sliding window and 20 kb step between the group of populations/varieties with unchanged seed color and those showing change in seed color using XP-CLR software v1.1.2 (https://github.com/hardingnj/xpclr) (Chen et al., 2010) and hapbin xpehh program version 1.3.0 (Maclean et al., 2015). The overlap of the top 1% of significant regions detected by the two methods considered as candidate regions and used to screen candidate genes according to the annotation file gff of *Phaseolus vulgaris* (https://ftp.ncbi.nlm.nih.gov/genomes/refseq/plant/Phaseolus_vulgaris/all_assembly_versions/GCF_000499845.1_PhaVulg1_0/). We selected the XP-CLR and XP-EHH tests for the detection of selection signatures because they complement each other in their strengths at moderate sample sizes. The XP-EHH test detects selective sweeps by identifying extended haplotype homozygosity and maintains robust statistical power even with limited sample sizes (Pickrell et al., 2009). Meanwhile, XP-CLR analyzes changes in the allele frequency spectrum between populations and has the advantage of providing high-resolution signals that allow precise identification of selected regions (Chen et al., 2010). The combination of these methods provides a robust framework for the detection of selection signatures in our dataset. To gain a better understanding of the gene functions and signaling pathways, we used g:GOSt in gProfiler (Raudvere et al., 2019) to test for overrepresentation of GO terms and Kyoto Encyclopedia of Genes and Genomes (KEGG) (Kanehisa et al., 2017) among the identified candidate genes. The statistical significance of over-represented GO terms within the input gene sets was assessed through Fisher’s exact tests, with a significance threshold set at False Discovery Rate (FDR) < 0.2.

## 3 Results

### 3.1 Morpho-phenotypic analysis of common bean composite populations

**Figure 1:**
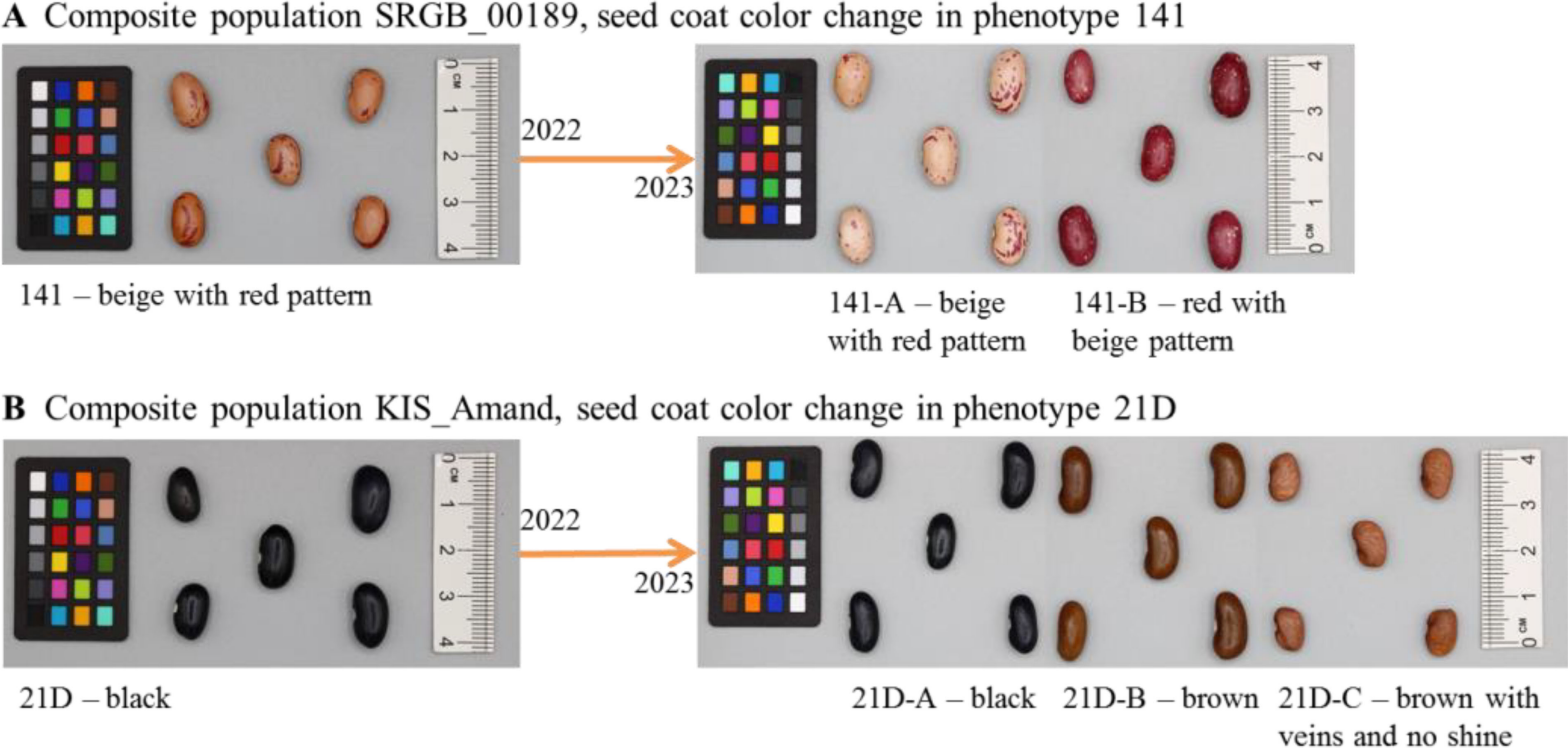
Examples of seed coat color change in composite population **(A)** SRGB_00189, phenotype 141 and **(B)** KIS_Amand, phenotype 21D.

**Figure 2:**
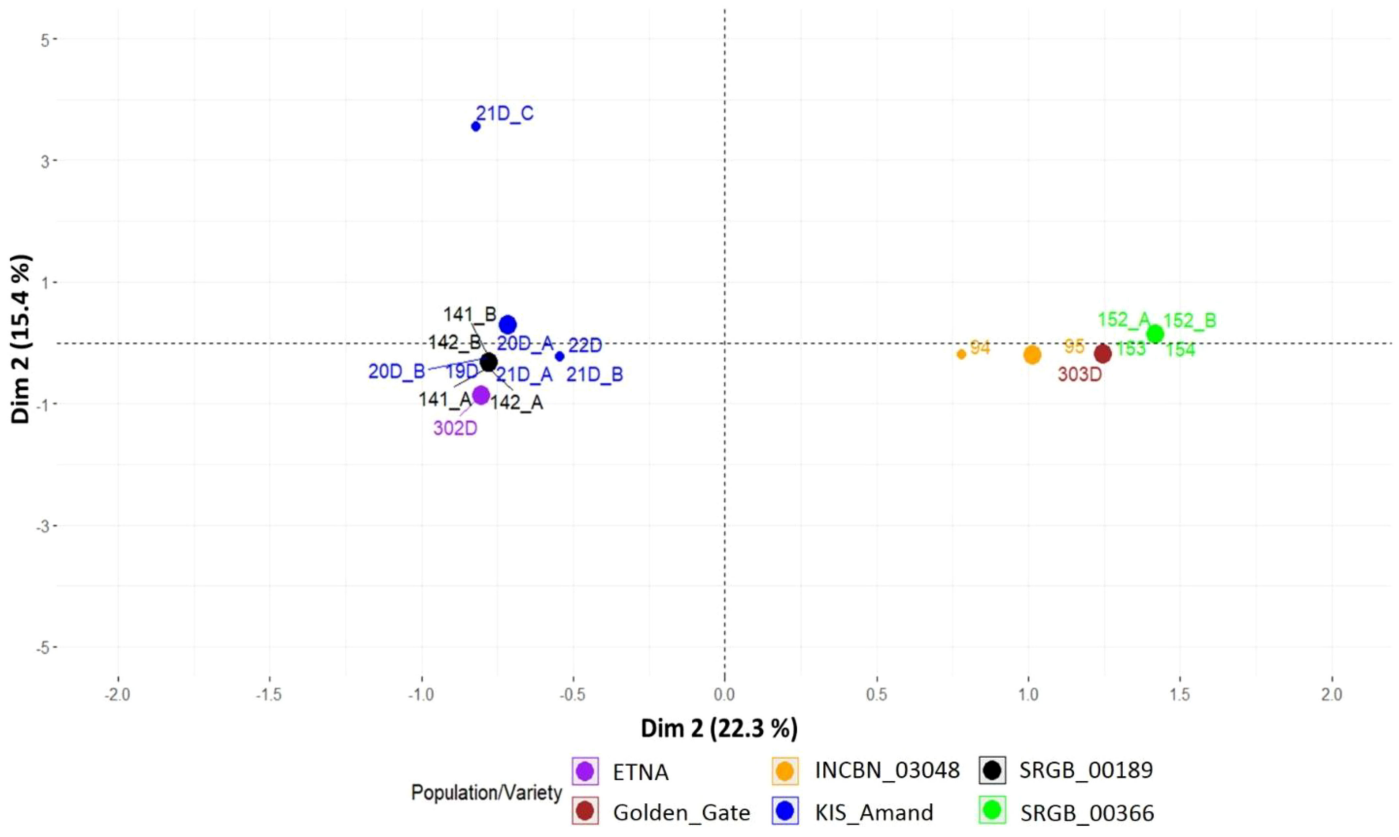
Morpho-phenotypic variation of composite populations and standard varieties of common bean based on multiple correspondence analysis.

Over the two years of phenotypic analysis, a wide diversity in seed coat color, growth habit and plant type was observed within and among the four composite populations. Notably, in 2023, accessions from KIS_Amand, SRGB_00189 and SRGB_00366 exhibited changes in seed coat color compared to 2022 (**Figure 1**). In contrast, no changes were observed in the composite population INCBN_03048 or in the standard varieties ETNA and Golden_Gate, which remained consistent across both years. The analysis of the patterns of the phenotypic variation in 2023 revealed that the composite populations exhibited a range of morphological differentiation, with KIS_Amand and SRGB_00366 displaying the most pronounced variability in seed coat color (SC), growth habit (GH), and plant type (PT). In contrast, the composite population SRGB_00189 showed greater homogeneity, especially in growth habit, which was predominantly determinate (**Table 1**). The Multiple Correspondence Analysis (MCA) of morpho-phenotypic variation revealed that the first two dimensions, Dim1 and Dim2, accounted for 22.3% and 15.4% of the total variation, respectively, highlighting significant phenotypic differences between the composite populations and varieties. The MCA biplot (**Figure 2**) divided the composite populations into two main groups along Dim1, with seed coat color emerging as the most prominent discriminating factor. KIS_Amand, SRGB_00189, and the standard variety ETNA formed the first group on the negative side of Dim1, characterized by dark primary seed coat colors such as black and brown. The second group, consisting of SRGB_00366, INCBN_03048 and Golden_Gate, was positioned on the positive side, distinguished by light seed coat colors such as white and beige. The positioning of the two standard varieties in the biplot suggests that they represent distinct morphological types within the bean spectrum, serving as reference points for assessing variation in the studied germplasm. Notably, SRGB_00366 and KIS_Amand displayed the widest distribution, indicating their greater morphological diversity. Growth habit also contributed significantly to the observed variation, with a clear distinction between determinate and indeterminate types, as well as between bush, prostrate, and climbing forms. SRGB_00366 was particularly notable for its ‘indeterminate prostrate growth with many lateral guides’, while other composite populations showed a mixture of determinate bush and indeterminate climber forms. Within the KIS_Amand population, the genotype 21D_C was notably distinguished by its unique seed coat color, described as ‘brown with veins and no shine’, and its ‘indeterminate bush with erect stems’ growth habit (**Figure 2**).

### 3.2 Genomic data and SNP annotation

**Figure 3:**
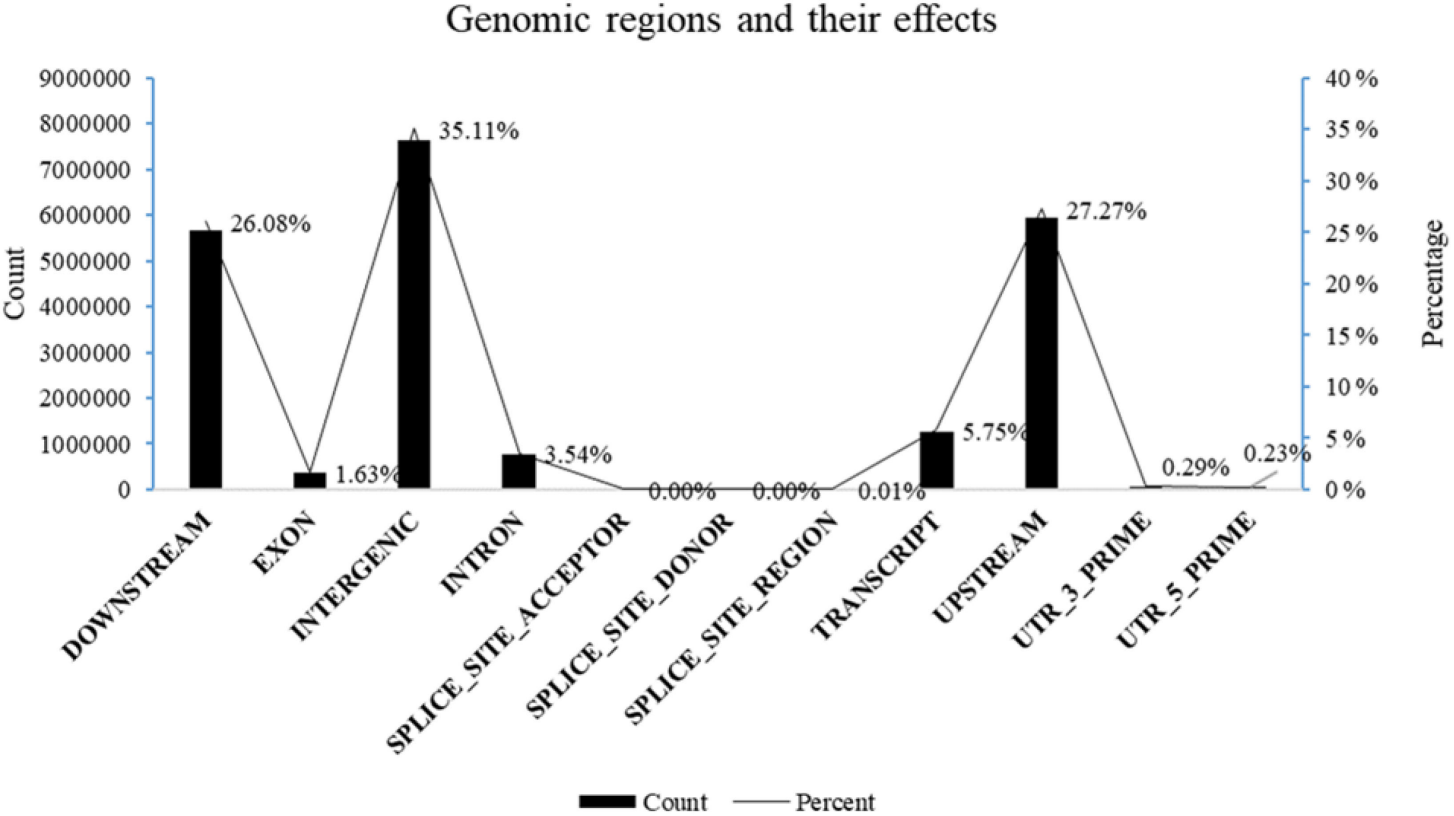
Distribution of SNPs in the genomic regions and their effects.

For the 19 individuals from the four composite populations and the two standard varieties of common bean, a total of 427 gigabytes of raw data were obtained for sequencing. This resulted in an average sequencing depth of approximately 30 × per individual, with an average Q30 value of 77% and an average guanine cytosine (GC) content of 37%. After quality control and filtering, the high-quality clean reads were mapped to the reference genome of common bean, achieving an average mapping rate of 97.71% and a mean coverage of 27.73 (**Supplementary Table 1**). We obtained 8,679,688 high-confidence single nucleotide polymorphism (SNP) sites, corresponding to an average density of 16.68 SNPs per kilobase in the *Phaseolus vulgaris* genome. The identified SNPs were physically mapped on the 11 chromosomes, with an overall distribution across the genome almost uniform. The highest number of SNPs (11%) were physically mapped on chromosomes four and one (999,032 and 980,805, respectively), with the highest mutation rates of 21.98 and 18.98 SNPs per kilobase, respectively. Chromosomes three and six exhibited the lowest number of mapped SNPs (7%; 568,520 and 567,652, respectively), while the lowest mutation rates were observed on chromosomes three and seven (11.11 and 14.07 SNPs per kilobase) (**Table 2**). The structural annotation of the identified SNPs showed that, based on their effect on the coding sequence, the largest proportion of SNPs was classified as a modifier (98.25% of SNPs affecting non-coding regions), followed by moderate (0.88% of SNPs could have a non-synonymous substitution), low impact (0.85% of SNPs with synonymous substitution) and the smallest value was recorded for SNPs with high impact (0.03% of SNPs with disruptive effects on the protein) (**Supplementary Table 2**). The distribution of SNPs across genomic regions was analyzed (**Figure 3**). SNP prevalence was highest in intergenic regions (35.11%), followed by upstream (27.28%) and downstream regions (26.08%), with 7,644,672, 5,939,851 and 5,678,805 SNPs, respectively. Introns (3.54%) and exons (1.63%) made up a smaller proportion with 771,068 SNPs and 354,491 SNPs. SNPs were less frequent in the 3’UTR (0.29%) and 5’UTR (0.23%) with 64,109 and 50,802 SNPs, respectively. In addition, splice site-related SNPs were rare, with 673 in the acceptor region of the splice site (0.003%), 514 in the donor region of the splice site (0.002%), and 19,152 in the splice site region (0.088%). The analysis revealed that most SNPs were located in non-coding regions. SNPs were further divided into heterozygous and homozygous variants, with homozygous variants outnumbering heterozygous variants in all samples analyzed, except for the three KIS_Amand phenotypes 20D_A, 20D_B and 21D_B. The effects by functional class revealed 159,882 SNPs (0.733%) being non-synonymous leading to amino acid changes. These substitutions mainly affected alanine (A), aspartic acid (D), glutamic acid (E) and leucine (L). However, as shown in **Supplementary Table 3**, these substitutions are not evenly distributed but concentrated on specific amino acid changes. These frequent changes could potentially impact protein function and stability, emphasizing the importance of these SNPs in altering protein properties. A number of amino acids exhibit a high frequency of self-substitutions, indicating regions where mutations do not change the amino acid sequence, a phenomenon known as synonymous mutation. Notable examples include alanine (12,742), glycine (12,698) and leucine (25,069), the latter of which demonstrates one of the most prevalent synonymous mutations within this dataset. Based on the nucleotide substitutions, among the identified SNPs, there were 204,665,968 transitions (Ts) and 103,693,961 transversions (Tv), corresponding to a Ts/Tv ratio of 1.97. Among the transitions, C/T substitutions were more frequent than G/A. Among transversions, C/A substitutions were more frequent compared to other combinations. In particular, the frequency of C/T transitions was significantly higher, reflecting their predominance over other types of transitions and transversions (**Supplementary Table 4**).

**Table 2:** Frequency distribution of SNPs discovered employing reference *Phaseolus vulgaris* L. genome of Andean common bean G19833 (GCF_000499845.1_PhaVulg1).

Chromosome	SNP Count	(%) of Total SNPs	Mutation rate (SNPs per kilobase)
Chr1	980805	11.30	18.98
Chr2	782040	9.01	16.15
Chr3	568520	6.55	11.11
Chr4	999032	11.51	21.98
Chr5	716074	8.25	17.74
Chr6	567652	6.54	14.69
Chr7	736906	8.49	14.07
Chr8	863629	9.95	18.79
Chr9	802003	9.24	17.86
Chr10	848873	9.78	15.48
Chr11	814154	9.38	16.59

### 3.3 Genetic diversity

The analysis of genetic diversity revealed clear patterns between composite populations and standard varieties (**Tables 3**, **4**). The composite population KIS_Amand had the highest nucleotide diversity and allelic richness (π = 0.222 and Ar = 1.380, respectively), reflecting considerable genetic variability, with 75.57% of loci polymorphic. However, the observed heterozygosity (Ho = 0.125) was significantly lower than the expected heterozygosity (He = 0.205), indicating possible inbreeding or unique population structure, as indicated by the high inbreeding coefficient (Fis = 0.364). In contrast, SRGB_00189 exhibited the lowest nucleotide diversity (π = 0.067 and Ar = 1.327, respectively) and a lower proportion of polymorphic sites (14.41%). Nevertheless, the observed heterozygosity (Ho = 0.097) was higher than the expected heterozygosity (He = 0.058), which could indicate recent population expansion or genetic admixture. SRGB_00189 exhibited an allelic richness (Ar = 1.327) relatively low compared to KIS_Amand, indicating lower genetic diversity at the allele level. SRGB_00366 had moderate nucleotide diversity (π = 0.173) and a moderate proportion of polymorphic sites (36.70%), with the observed heterozygosity (Ho = 0.229) exceeding the expected heterozygosity (He = 0.150). This indicates a balanced genetic diversity supported by the allelic richness (Ar = 1.338), which is intermediate among the composite populations. INCBN_03048 had a nucleotide diversity of 0.124, indicating moderate genetic variation, with 20.27% of loci being polymorphic. The observed heterozygosity (Ho = 0.159) was higher than the expected heterozygosity (He = 0.092). The allelic richness for INCBN_03048 was the lowest among the composite populations (Ar = 1.047), suggesting lower allelic diversity. Among standard varieties, Golden_Gate showed π and He, Ar of 0.215, 0.108, and 1.060, respectively, indicating considerable genetic diversity, with a Ho of 0.215 and 21.54% of polymorphic sites. In contrast, ETNA with an Ar of 1.059 has a lower π of 0.094, a lower proportion of polymorphic sites (9.41%), and lower observed and expected heterozygosity of 0.094 and 0.047, respectively. The Fis value of 0 indicates a balance in inbreeding, but the reduced π value emphasizes a lower level of overall genetic diversity.

**Table 3:** Genetic diversity parameters among the composite populations and standard varieties of common bean.

CPOP/VAR	Num_Indv	Ar	Private Sites	Variant Sites	Polymorphic Sites	Polymorphic sites (%)	Obs_Ho	Exp_Ho	Ho	He	π	Fis
KIS_Amand	7	1.380	664125	8453390	6387768	75.57	0.875	0.795	0.125	0.205	0.222	0.364
SRGB_00189	4	1.327	51499	8341341	1201770	14.41	0.903	0.942	0.097	0.058	0.067	-0.054
SRGB_00366	4	1.338	424490	8318775	3053167	36.70	0.771	0.850	0.229	0.150	0.173	-0.095
INCBN_03048	2	1.047	122150	8277391	1678127	20.27	0.841	0.908	0.159	0.092	0.124	-0.053
Golden_Gate	1	1.060	135222	8170944	1759611	21.54	0.785	0.892	0.215	0.108	0.215	0
ETNA	1	1.059	32922	8298175	780855	9.41	0.906	0.953	0.094	0.047	0.094	0

CPOP, composite population; VAR, standard variety; Num_Indv, number of individuals.

**Table 4:** Fst values among the composite populations and standard varieties of common bean.

	KIS_Amand	SRGB_00189	SRGB_00366	INCBN_03048	ETNA	Golden_Gate
KIS_Amand	0.000					
SRGB_00189	0.085	0.000				
SRGB_00366	0.383	0.610	0.000			
INCBN_03048	0.219	0.573	0.456	0.000		
ETNA	0.052	0.134	0.584	0.586	0.000	
Golden_Gate	0.268	0.679	0.279	0.569	0.704	0.000

### 3.4 Runs of homozygosity and genomic inbreeding

**Figure 4:**
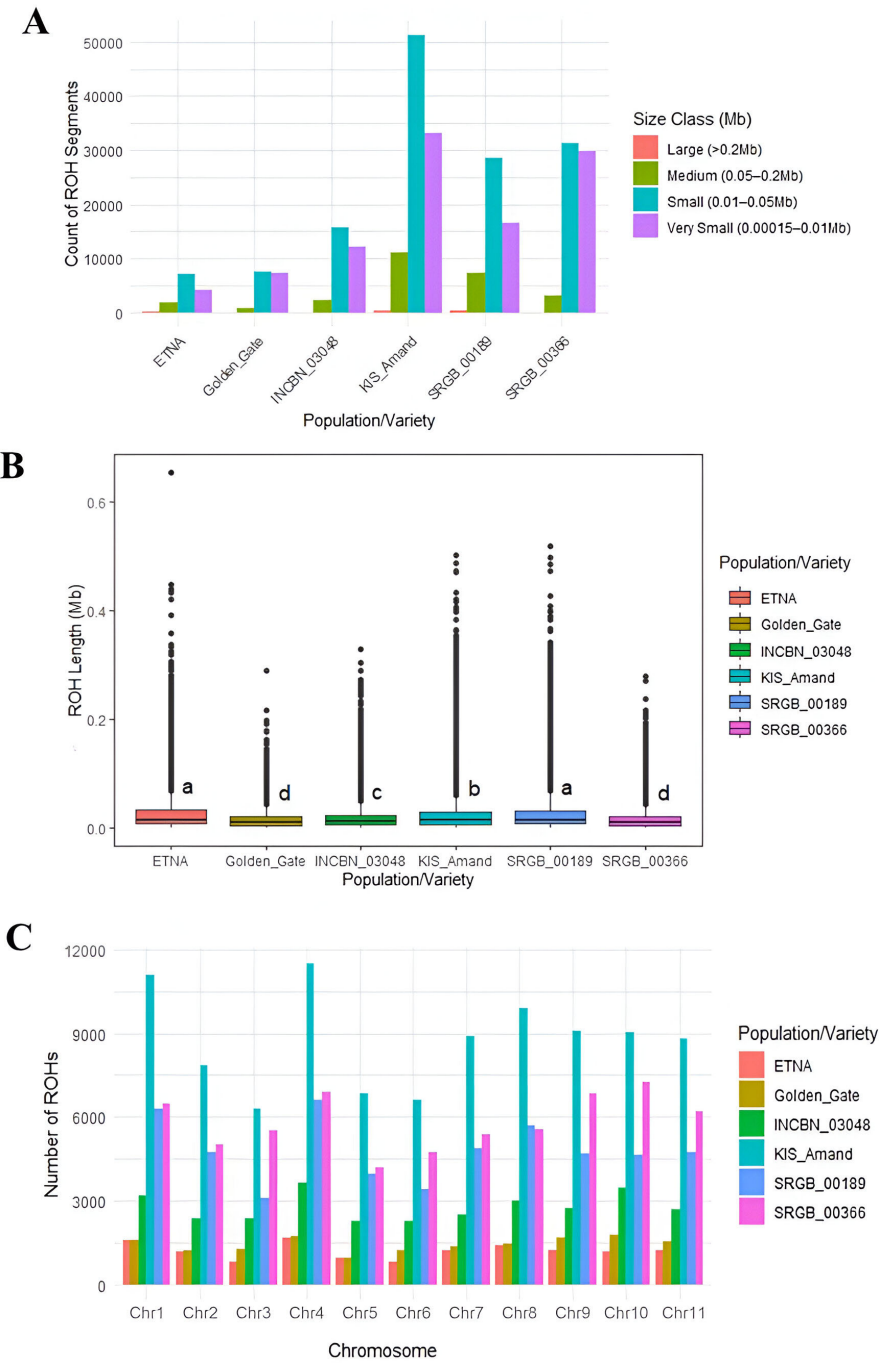
**(A)** Distribution of ROH lengths in each composite population/standard variety, **(B)** mean ROH length in the studied composite populations and standard varieties, lowercase letters indicate composite populations with equal medians, and **(C)** distribution of the total number of ROHs per chromosome.

Using the sliding window approach, a total of 271,508 runs of homozygosity (ROH) segments in four composite populations and two standard varieties were identified. The analysis revealed that most ROHs detected in the composite populations and varieties were small to very small. Among all ROHs identified, 830 large ROHs (> 0.2 Mb) were observed, mainly in KIS_Amand (353 ROHs) and SRGB_00189 (340 ROHs). We also identified 26,440 medium-sized ROHs (0.05 – 0.2 Mb), 141,111 small ROHs (0.01 – 0.05 Mb), and 103,127 very small ROHs (0.00015 – 0.01 Mb) (**Figure 4A**). The longest ROH segment was detected in the standard variety ETNA with a size of 0.65 Mb and 9,268 SNPs on chromosome four while the shortest one was observed in SRGB_00189 with a size of 0.000151 Mb and 56 SNPs on chromosome one. A comparative analysis of the mean ROH lengths revealed significant differences between the four composite populations and the standard varieties. The standard variety ETNA had the highest mean ROH length of 0.028 Mb, followed by the composite populations SRGB_00189 with 0.027 Mb and KIS_Amand with 0.024 Mb (**Figure 4B**). These results indicated an increased level of inbreeding within the ETNA, as longer ROH segments are typically associated with lower genetic diversity and increased homozygosity. The distribution of the total number of ROHs in the studied composite populations and varieties, as well as the distribution per chromosome was presented in **Figures 4A, C**, and **Supplementary Table 5**. The highest number of ROHs was observed in KIS_Amand, with 11,505 on chromosome four and 11,087 on chromosome one. In contrast, the standard varieties ETNA and Golden_Gate had the lowest number of ROHs, ranging from 786 in ETNA on chromosome three to 1,750 in Golden_Gate on chromosome ten. On average, the number of ROHs per population was highest in KIS_Amand (8,709), followed by SRGB_00366 (5,811), SRGB_00189 (4,788), and INCBN_03048 (2,754). For the two standard varieties, the average number of ROHs was 1,194 for ETNA and 916 for Golden_Gate.

### 3.5 Population differentiation and genetic structure

**Figure 5:**
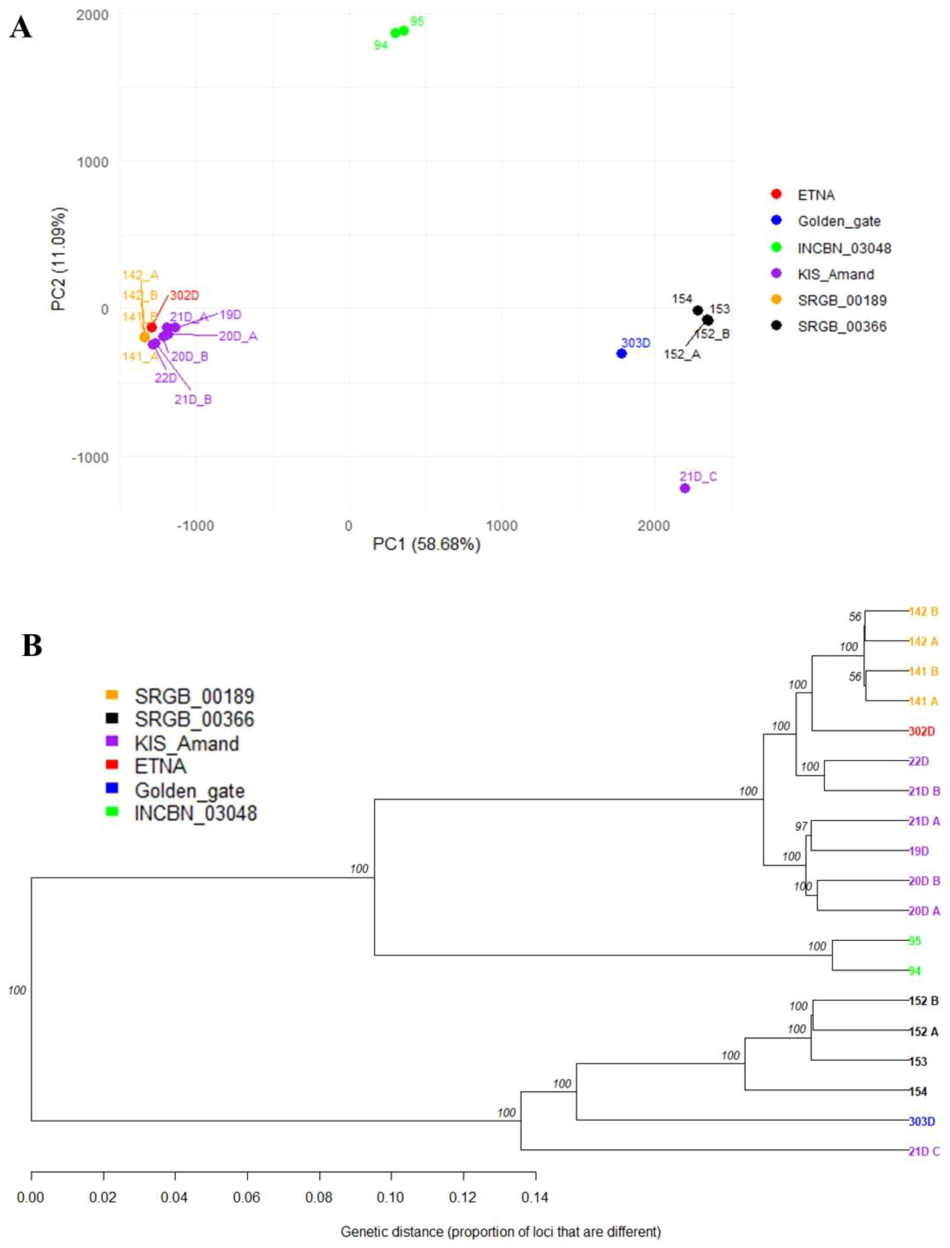
The genetic structure of composite populations and standard varieties of common bean based on **(A)** PCA and **(B)** UPGMA cluster analysis.

The differentiation among the four composite populations and the two standard varieties was analyzed, and a strong genetic divergence (Fst = 0.704) between the standard varieties was revealed. These varieties were also notably different from the composite populations, except for the relatively close relationship between KIS_Amand and ETNA (Fst = 0.052), with Fst values ranging from 0.134 (ETNA – SRGB_00189) to 0.679 (Golden_Gate – SRGB_00189). A strong genetic divergence was also found among pairs of the composite populations, with the highest divergence between SRGB_00366 and SRGB_00189 (Fst = 0.610) while KIS_Amand and SRGB_00189 were the most genetically close to each other (Fst = 0.085). The genetic structure and relationships among the four composite populations and the two standard varieties were further analyzed using principal component analysis (PCA) and clustering analyses. PCA showed that the two first principal components explained 69.77% of the total variation with PC1 explaining most of the variation (58.68%), indicating a strong genetic differentiation along this axis. As shown in **Figure 5A**, PCA revealed a clear clustering pattern, indicating that individuals within a composite population tend to group and are distinctly separated from individuals in other populations. Samples from SRGB_00366 and INCBN_03048 formed two independent groups on the positive side of PC1, indicating relatively isolated genetic profiles. In contrast, KIS_Amand and SRGB_00189 appeared grouped on the negative side of PC1, potentially reflecting shared ancestry. This clustering pattern is consistent with the results of MCA and may be associated with growth habit of the plants. The PCA plot further indicated that Golden_Gate was genetically closer to SRGB_00366, while ETNA was closer to KIS_Amand and SRGB_00189. Interestingly, the first group (Golden_Gate and SRGB_00366) exhibited an indeterminate growth habit, while the second group (ETNA, KIS_Amand and SRGB_00189) displayed a determinate growth habit. This grouping pattern was also confirmed by the hierarchical clustering analysis with very high bootstraps (**Figure 5B**).

### 3.6 Genetic background of seed coat color change

**Figure 6:**
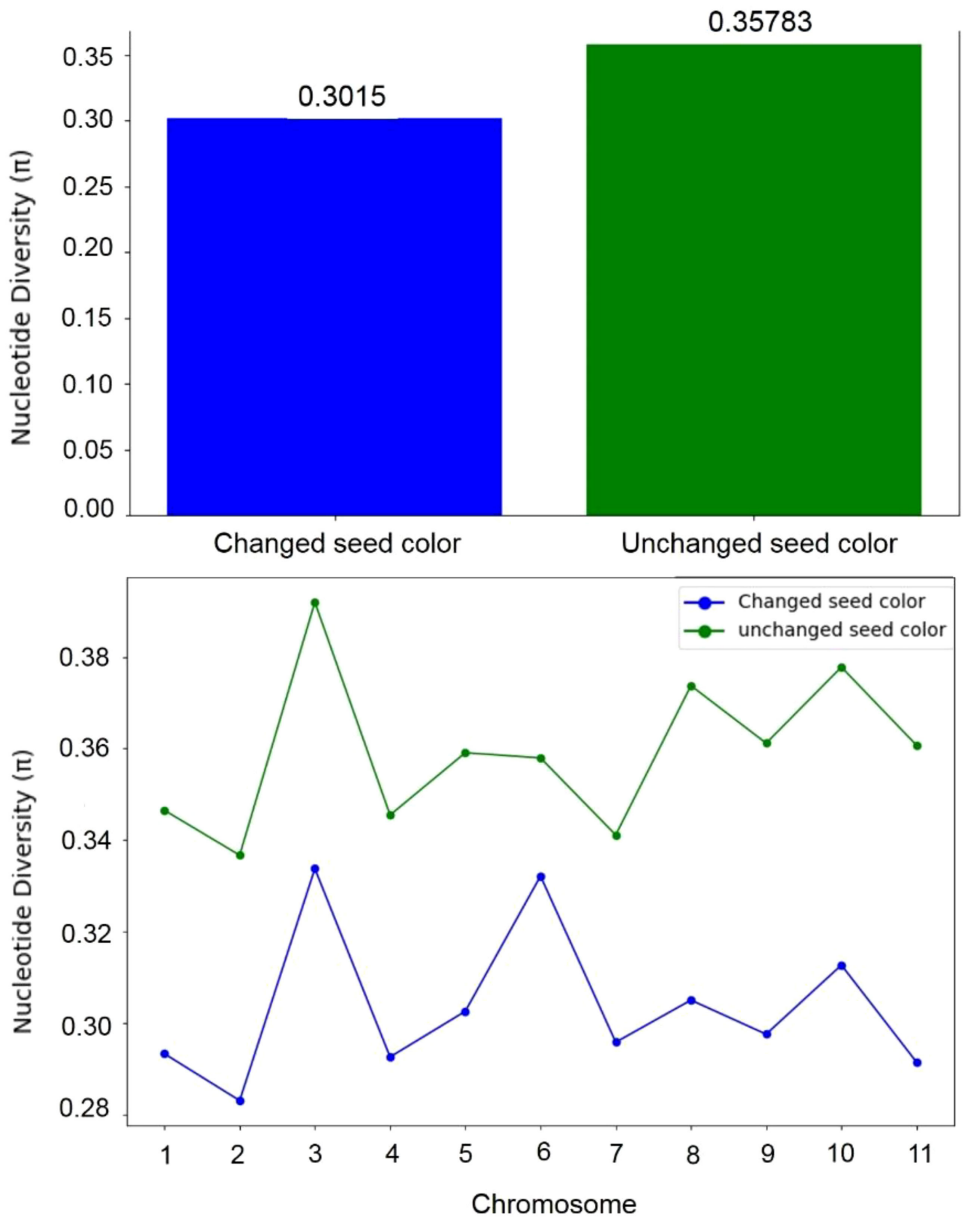
Nucleotide diversity (π) in populations with altered seed coat color and populations with unchanged seed coat color.

**Figure 7:**
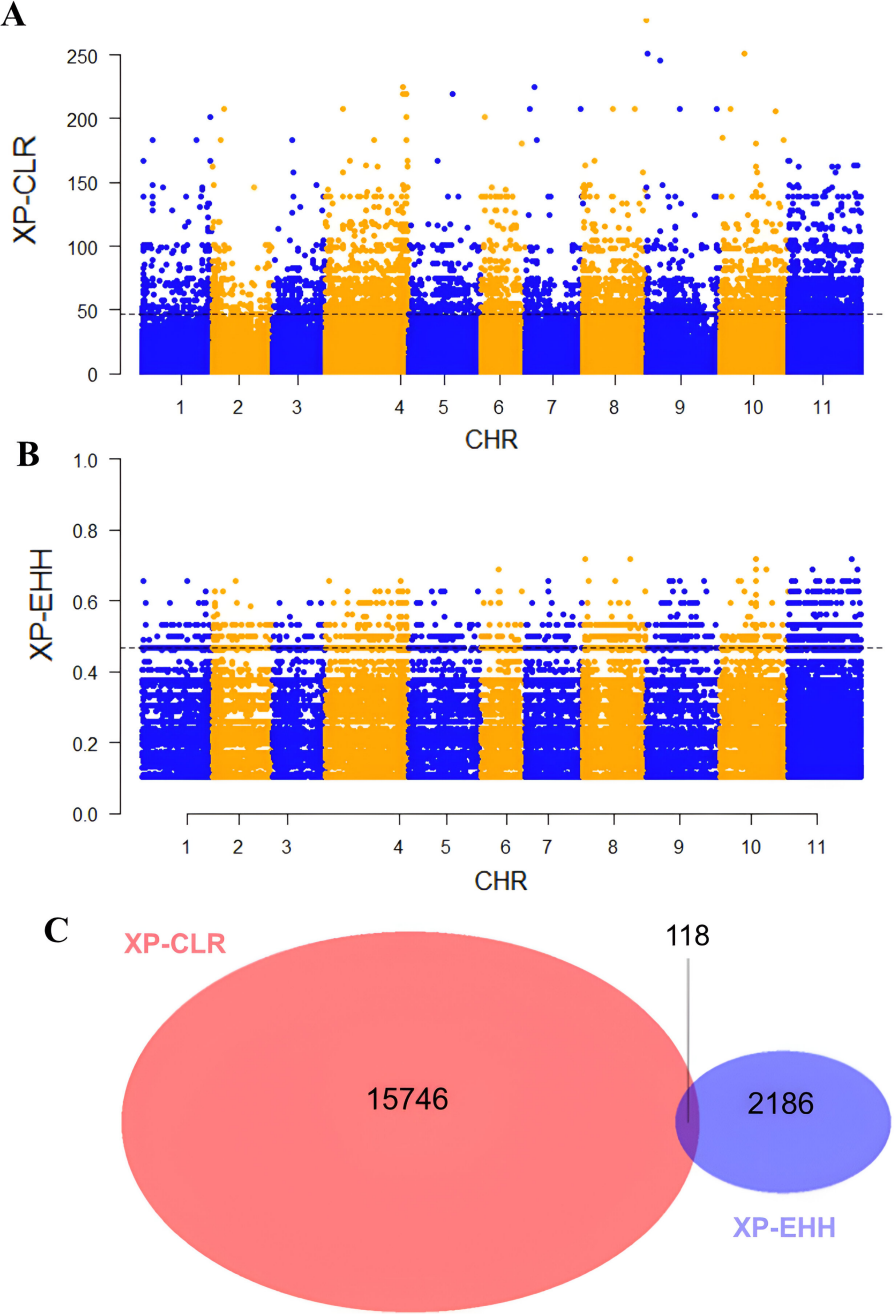
Manhattan plots of **(A)** pairwise XP-CLR and **(B)** XP-EHH showing the signatures of positive selection between populations with altered seed coat color and unaltered seed coat color. The top 1% of the empirical distribution of XP-CLR and XP-EHH scores are indicated by dotted lines, respectively. **(C)** Venn diagrams showing the overlap between the genomic regions identified by XP-CLR and XP-EHH tests.

**Figure 8:**
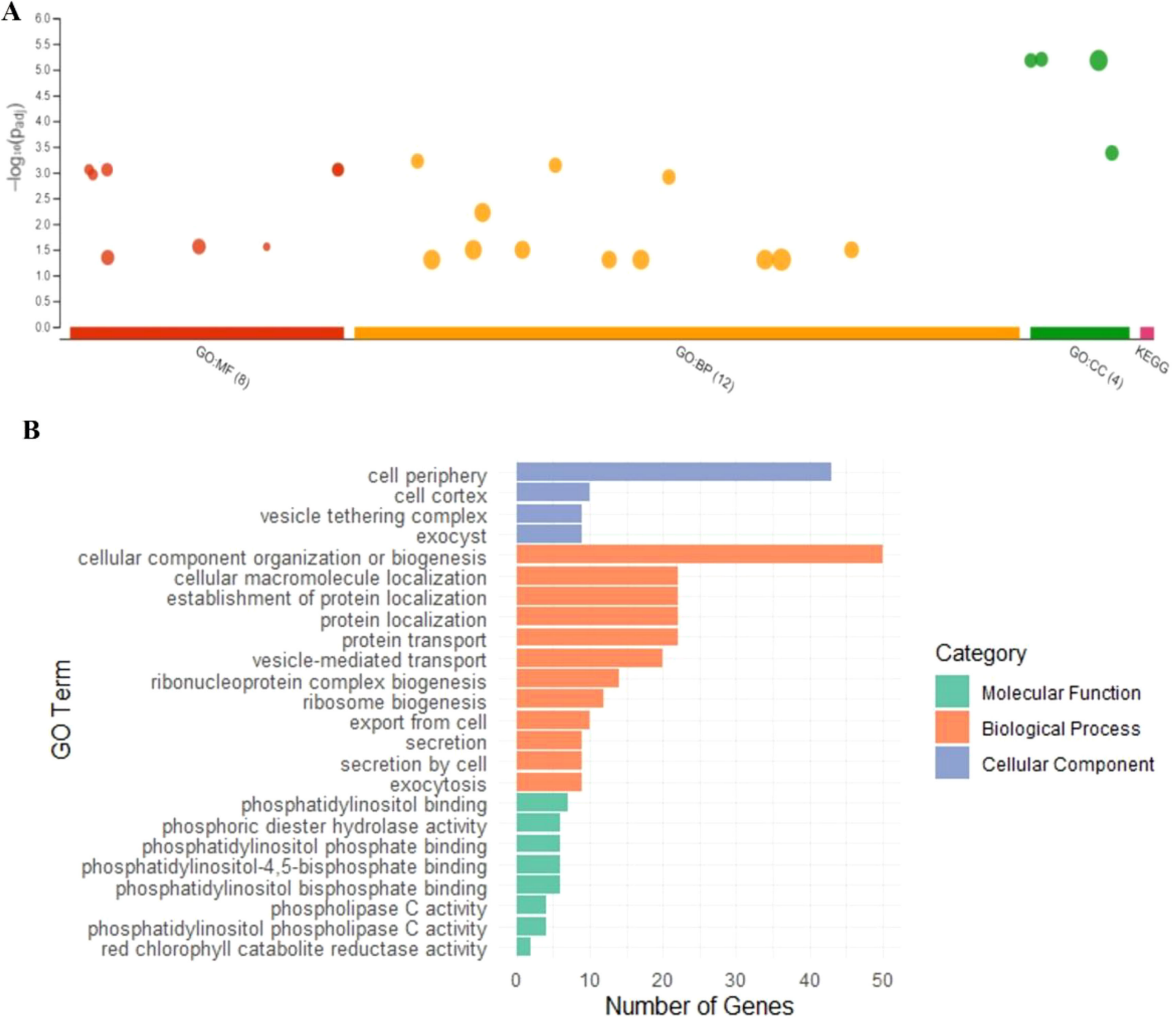
**(A)** Overrepresented functions of genes under pervasive positive selection (*P* < 0.05) and **(B)** enrichment categories of regions under positive selection.

To assess whether seed coat color was subject to selection during the evolution of the studied materials, we compared nucleotide diversity (π) between the composite populations that experienced a change in seed coat color and those that maintained their original seed coat color. Our analysis revealed lower nucleotide diversity (π) in the populations that underwent seed coat color change compared to those that did not. Across all chromosomes, π averaged 0.30 in the seed coat color change group and 0.36 in the other group, reflecting a 15.69% reduction. This decrease in diversity suggests the presence of a selective signature associated with the seed coat color change (**Figure 6**). To accurately identify the genomic regions associated with seed color change, we performed a genome-wide scan for evidence of selection signals. We used two statistics, XP-EHH and XP-CLR, to compare populations with altered seed color to those with unaltered seed coat color. The top 1% of significant regions detected by the two methods were used as candidate regions. Using the XP-CLR test (**Figure 7A**), a total of 15,746 significant regions (XP-CLR > 47) were detected, with chromosome four having the highest number of regions (4,419), followed by chromosomes eleven, ten and five (2,977, 1,722 and 1,417 regions, respectively). Using the XP-EHH test, 2,186 significant regions were detected (XP-EHH > 0.47) (**Figure 7B**), with chromosomes eleven and four containing the most regions (908 and 306, respectively) (data not shown). Among all significant regions found, 118 regions shared by both methods located mainly on chromosomes four, nine, ten, and eleven were therefore considered candidate regions for further analysis (**Figure 7C**). Gene Ontology (GO) and KEGG enrichment analyses performed with g:profiler to explore the candidate regions’ biological functions revealed the significant enrichment of 101 gens and several highly enriched functional categories belonging to 24 GO terms. In the molecular function category, eight GO terms were particularly enriched, including phosphatidylinositol bisphosphate binding (GO:1902936), phosphatidylinositol phospholipase C activity (GO:0004435), the binding of phosphatidylinositol 4,5-bisphosphate (GO:0005546), the binding of phosphatidylinositol phosphate (GO:1901981) and the activity of phospholipase C (GO:0004629), with a *P*-value of < 0.01. For biological processes, twelve GO terms showed significant enrichment, with exocytosis (GO:0006887), secretion by the cell (GO:0032940), secretion (GO:0046903), and vesicle-mediated transport (GO:0016192) being the most significant. For the cellular component, GO terms such as cell periphery (GO:0071944), cell cortex (GO:0005938), exocyst (GO:0000145), and vesicle tethering complex (GO:0099023) were identified as highly significant, with a *P*-value of < 0.001 (**Table 5**; **Figure 8**).

**Table 5:** GO terms obtained by enrichment analysis of the candidate regions.

GO term	Description	Ontology	Adjusted P value
GO:1902936	phosphatidylinositol bisphosphate binding	MF	0.001
GO:0004435	phosphatidylinositol phospholipase C activity	MF	0.001
GO:0005546	phosphatidylinositol-4,5-bisphosphate binding	MF	0.001
GO:1901981	phosphatidylinositol phosphate binding	MF	0.001
GO:0004629	phospholipase C activity	MF	0.001
GO:0035091	phosphatidylinositol binding	MF	0.027
GO:0051743	red chlorophyll catabolite reductase activity	MF	0.027
GO:0008081	phosphoric diester hydrolase activity	MF	0.044
GO:0006887	exocytosis	BP	0.001
GO:0032940	secretion by cell	BP	0.001
GO:0046903	secretion	BP	0.001
GO:0016192	vesicle-mediated transport	BP	0.006
GO:0015031	protein transport	BP	0.030
GO:0022613	ribonucleoprotein complex biogenesis	BP	0.030
GO:0140352	export from cell	BP	0.030
GO:0071840	cellular component organization or biogenesis	BP	0.046
GO:0008104	protein localization	BP	0.046
GO:0042254	ribosome biogenesis	BP	0.046
GO:0045184	establishment of protein localization	BP	0.046
GO:0070727	cellular macromolecule localization	BP	0.046
GO:0071944	cell periphery	CC	0.000
GO:0005938	cell cortex	CC	0.000
GO:0000145	exocyst	CC	0.000
GO:0099023	vesicle tethering complex	CC	0.000

BP, biological process; MF, molecular function; CC, cell component.

## 4 Discussion

This study represents the first genomic characterization of composite populations of the common bean. Given their diverse origin and phenotypic seed characteristics, these populations represent a unique set with unknown traits and potential advantages, particularly in the context of organic breeding and climate change. The study included four composite populations of common bean, comprising 17 individual phenotypes that exhibited the highest level of segregation following the initial year of field evaluation, as well as two standard varieties. The two-year phenotypic analysis revealed considerable diversity in seed coat color within and among the four composite populations, with notable differences in growth habit and plant type observed among populations. In 2023, changes in seed coat color were observed in the populations KIS_Amand, SRGB_00189 and SRGB_00366, whereas population INCBN_03048 and the standard varieties ETNA and Golden_Gate remained phenotypically stable in both years. The results of multiple correspondence analyses (MCA) indicated that seed coat color was a significant discriminating factor, with the populations clustering into two main groups based on their dark or light seed coat. The highest degree of variability was observed in KIS_Amand and SRGB_00366, while SRGB_00189 exhibited more uniform characteristics, particularly in regard to growth habit. The changes in seed coat color observed in KIS_Amand, SRGB_00189 and SRGB_00366 between years 2022 and 2023 suggest the potential influence of genetic or environmental factors. In contrast, the stability of seed coat color in INCBN_03048 and both standard varieties ETNA and Golden_Gate suggests the possibility of genetic resistance to phenotypic shifts. These findings underscore the significance of investigating the genetic basis of seed coat color variation, as this may elucidate key adaptive traits for incorporation into breeding programs. The variability observed in common bean composite populations can be attributed to a number of factors, including mutations, genetic drift, environmental conditions, and local adaptations, which can lead to an increase in the outcrossing rate. Historical landraces have demonstrated notable shifts in genetic and phenotypic traits after only a few generations in differential environments, in part due to gene flow from proximate varieties (Klaedtke et al., 2017). While ecological and geographical factors contribute to differentiation, evidence suggests that a significant portion of seed coat color variation in common beans is genetically determined, indicating the potential for targeted optimization through selection (Bassett, 2007; McClean et al., 2018; García-Fernández et al., 2021; Parker et al., 2024).

Building on these findings, we conducted whole-genome sequencing to generate a comprehensive genetic and genomic dataset for the four composite populations and the two standard varieties of *Phaseolus vulgaris*. This analysis revealed a high level of genetic diversity, providing previously unavailable insights into the genomic architecture and evolutionary history of the species. The high-quality sequencing data (427 GB) and depth of coverage (30 ×) represent a significant advancement over previous studies in common bean genomics (Raggi et al., 2019; García-Fernández et al., 2021), facilitating the identification of subtle genetic variations that may have been previously overlooked. The remarkably high mapping rate (97.71%) to the reference genome indicates robust genomic conservation across populations, despite observed phenotypic variation. The distribution of over 8.6 million high-confidence SNPs across the genome provides a detailed representation of the genetic variation observed in the studied germplasm. The observed SNP density (16.68 SNPs per kilobase) is consistent with the general levels of genetic variation reported in previous studies on common bean populations. Additionally, distinct chromosomal patterns in SNP density were observed, potentially reflecting the evolutionary history and population dynamics of these populations (Raggi et al., 2019; Delfini et al., 2021). The higher concentrations of SNPs on chromosomes four and one in comparison to the lower densities on chromosomes three and six indicate differential selection pressure across the genome. These patterns may be the consequence of historical breeding practices or natural selection acting on specific genomic regions associated with agronomically important traits. In accordance with the findings of Valdisser et al. (2017) regarding a Brazilian core collection of common bean, our study similarly revealed a prevalence of SNPs in non-coding regions, offering valuable insights into the regulatory architecture of the common bean genome. The prevalence of variants in intergenic, upstream and downstream regions indicates that adaptations may be predominantly attributable to changes in gene regulation rather than alterations in protein-coding sequences (Li et al., 2023). The structural categorization of SNPs as predominantly modifiers with limited high-impact variants reflects a balance between conservation and innovation within the genome. Moreover, the observed transition-to-transversion ratio of 1.97, coupled with the prevalence of C/T transitions, provides insights into the mutational mechanisms influencing these populations. This findings are in accordance with those reported by Valdisser et al. (2017) for a Brazillian common bean collection, Gelaw et al. (2023) for an Ethiopian collection, and Lioi et al. (2019) for an Italian common bean collection.

Furthermore, despite the relatively limited sample size (1 – 7 individuals per population), we found considerable levels of genetic diversity (He = 0.047 – 0.205), comparable to those reported in larger studies on domesticated common bean collections, such as those by Valdisser et al. (2017) (He = 0.168, n = 111) and Rodriguez et al. (2016) (He = 0.157, n = 100). Even in smaller but phenotypically diverse collections, such as the one studied by Cichy et al. (2015) (He = 0.233, n = 21), the genetic diversity observed here is consistent with the broader diversity across common bean varieties. Interestingly, the genetic diversity patterns observed in this study revealed significant correlations between genomic variation and seed coat color change across diverse populations. Indeed, the composite populations KIS_Amand and SRGB_00366, which both exhibited seed coat color change, demonstrated the highest levels of nucleotide diversity (π) and allelic richness (Ar), which align with their high morpho-phenotypic diversity. This suggests that the genetic diversity observed in these populations is associated with their capacity to manifest diverse phenotypes, such as variations in seed coat color. The elevated genetic variation observed in these populations is likely to facilitate their adaptability to different selection pressures, whether occurring naturally or as a result of human intervention. These patterns may be influenced by agricultural practices, whereby the selection of traits such as seed coat color may be combined with the preservation of genetic diversity at other loci, thereby promoting a wide range of adaptive potential (Khoury et al., 2022; Alam and Purugganan, 2024). Conversely, population SRGB_00189, which exhibited a change in seed coat color, showed a discrepancy between the observed heterozygosity (Ho = 0.097) and the expected heterozygosity (He = 0.058). This was accompanied by a negative Fis value, indicating an excess of heterozygotes within the population. Two distinct patterns are observed in the seed coat coloration. The primary pattern exhibits darker mottling on a lighter-colored background, while the alternate pattern reverses this, with the background appearing darker and the mottling lighter. This patterning appears to be under the control of the *C* locus. Specifically, a heterozygous form of the *C* gene (*Cc*) exerts an influence on the pattern, with additional modifier genes within the *C* locus affecting the specific appearance of the pattern. This type of seed coat variation is observed at low frequencies in the majority of bean cultivars with *C*-locus patterns (Bassett, 2007). The heterozygote excess and distinctive seed coat pattern observed in SRGB_00189 may be indicative of genetic drift or a distinctive demographic history, such as population bottlenecks or admixture events. Such events could have resulted in the preservation of heterozygosity in specific genomic regions (such as the *C* locus) while leading to genetic uniformity (fixation) in others. The lower nucleotide diversity in SRGB_00189 (π = 0.067) provides further evidence that this population may have undergone recent population expansions or been subject to genetic drift, which may have impacted loci controlling seed coat color, leading to phenotypic fixation. It is noteworthy that the two standard varieties, ETNA and Golden_Gate, which also exhibited minimal variation in seed coat color, demonstrated comparable levels of genetic diversity (He = 0.047 – 0.108). The low nucleotide diversity and fixed phenotypes observed in these varieties are likely the result of intensive selective breeding, where uniformity in traits such as seed coat color is prioritized, potentially at the cost of broader genomic variation. This lends support to the notion that rigorous selection for particular traits, such as seed coat color, can result in a reduction of genetic diversity across the genome. Moreover, previous studies have shown that high-frequency ROH regions shared by individuals frequently indicate the presence of selection pressure in populations (Pemberton et al., 2012; Caivio-Nasner et al., 2021). In our study, the composite population SRGB_00189 and the standard variety ETNA, both of which exhibited low genetic diversity and a stable seed coat color, demonstrated the highest ROH coverage. This indicates that there has been a significant focus on selective breeding with the objective of maintaining specific traits, including seed coat color. In contrast, the extensive ROHs observed in KIS_Amand indicate the potential for inbreeding and selection pressure, particularly in relation to the observed phenotypic changes in seed coat color. Furthermore, the genetic structure analysis yielded a clustering pattern that was consistent with the morpho-phenotypic data, thereby reinforcing the assumption that seed coat color variation is predominantly driven by genetic factors. These findings highlight the pivotal role of selection in shaping both the genetic and phenotypic diversity within the analyzed germplasm. The differential nucleotide diversity (π) between populations with altered and stable seed coat color provides compelling evidence that selection is acting on seed coat color-related genomic regions in common bean. The 15.69% reduction in diversity (π = 0.30 vs 0.36) observed in populations displaying alterations in seed coat color is consistent with selective sweep patterns, whereby positive selection results in a reduction in genetic variation within regions linked to advantageous alleles. Similar findings have been documented in chickpea, where a 61% reduction in nucleotide diversity was observed in light brown/yellow-brown ‘desi’ chickpea accessions relative to ‘kabuli’ accessions with beige seed coats. This highlights the influence of strong purifying selection on seed coat color (Bajaj et al., 2015). Comparable signatures of selection have also been documented in chickpea (Varshney et al., 2013) and in rice (Sweeney et al., 2007), which further emphasizes the evolutionary pressures shaping seed color variation across crop species.

It is important to acknowledge that evolutionary and demographic processes, such as the bottleneck effect at the outset of breeding and the different effective population sizes throughout the improvement process, can give rise to discrepancies in genetic composition among populations. This, in turn, may give rise to false positives (Zhang et al., 2024). However, the combination of XP-EHH and XP-CLR methods employed in this study effectively mitigates the confounding effects. This robust analytical approach enabled the identification of divergent genomic regions that are likely to harbor candidate genes or loci associated with phenotypic changes in seed coat color within the studied populations. Previous research has successfully utilized these methods to detect significant genomic regions in various legumes, including common bean (M Rendón-Anaya et al., 2023) and soybean (Zuo et al., 2022; Cho et al., 2024). Notably, XP-EHH is particularly adept at identifying selective genomic regions even with small sample sizes (Pickrell et al., 2009), thereby enhancing the reliability of our analysis and strengthening our confidence in the identified candidate regions. For example, Zhang et al. (2021) used 39 samples, while Kumar et al. (2020) used only nine samples. Our sample size is also consistent with several pivotal studies on common bean genomics, including Bitocchi et al. (2017b; n=45), which have made important contributions to the field.

Previous studies have shown that seed coat color in legumes is predominantly determined by the accumulation of phenolic compounds, including flavonoids, phenolic acids, and proanthocyanidins, which are synthesized through the shikimate/phenylpropanoid pathway and specifically accumulate in the seed coat tissue (Ranilla et al., 2007; Singh et al., 2017). While traditional genetic studies in common bean have focused on genes directly controlling pigment biosynthesis and spatial distribution (Bassett, 2007; Parker et al., 2024), our results demonstrated that there is an additional layer of complexity in determining seed coat color. Using complementary selection scans (XP-EHH and XP-CLR) led to the identification of a total of 118 candidate regions were identified, 29 of which exhibited a high degree of enrichment within the phosphatidylinositol pathway. These findings indicate that cellular transport mechanisms may be a significant factor influencing the observed variation in seed coat coloration. The connection between phosphatidylinositol signaling and pigmentation is consistent with findings from other legumes, where anthocyanin production is induced via the phosphatidylinositol 3-kinase/Akt pathway in black soybean seed coats (Tsoyi et al., 2008) and phosphatidylinositol 4-phosphate 5-kinase is upregulated in faba bean seed coats (Ray et al., 2015). The significant enrichment of phosphatidylinositol-related functions and vesicle-mediated transport (GO:0016192) in the identified selection signatures indicates the presence of a coordinated system in which the cellular transport machinery works in conjunction with pigment biosynthesis to determine seed coat color phenotypes (Wan et al., 2016; Ren et al., 2017; Campa et al., 2023).

## 5 Conclusion

The integration of high-coverage whole-genome sequencing with detailed phenotypic analysis in this study has revealed novel insights into the genetic architecture of seed coat color variation in common bean. The identification of 8.6 million high-confidence SNPs revealed distinct patterns of genetic diversity across composite populations. It is noteworthy that KIS_Amand exhibited the highest nucleotide diversity (π = 0.222) and allelic richness (Ar = 1.380), which contrasts sharply with the limited diversity observed in SRGB_00189 (π = 0.067, Ar = 1.327). This suggests that the two populations have undergone divergent evolutionary trajectories due to different selection pressures. Our analysis revealed two key findings regarding population structure and selection. First, the strong genetic differentiation between standard varieties (ETNA and Golden_Gate, Fst = 0.704) and composite populations illustrates the profound impact of selective breeding on genetic architecture. Second, the reduced genetic diversity in populations with modified seed coat colors points to ongoing positive selection, particularly evident in the 118 selective sweeps identified primarily on chromosomes four, nine, ten and eleven. The pathway analysis revealed a critical role for phosphatidylinositol signaling in seed coat pigmentation. This finding extends beyond the traditional pigment biosynthesis pathways to implicate cellular transport mechanisms in color development, suggesting a more complex regulatory network than was previously recognized. The enrichment of genes involved in vesicle-mediated transport (*P* < 0.001) and exocytosis (*P* < 0.01) within selective sweep regions provides substantial support for this novel mechanistic model. These findings have direct implications for the development of breeding programs. The identified selective sweeps provide precise genomic targets for breeding programs focused on seed coat color modification. The high diversity observed in composite populations such as KIS_Amand represents a valuable genetic resource for maintaining crop resilience while improving aesthetic traits. The newly discovered role of transport pathways offers alternative approaches of modifying seed coat color characteristics, extending beyond the scope of traditional pigment pathway manipulation. With further aim of validating the identified regions and conducting transcriptomic analysis to better understand the underlying regulatory mechanisms, this research advances both theoretical understanding and practical applications in common bean improvement, thereby contributing to broader goals of crop enhancement and agricultural sustainability.

## Data Availability

The datasets presented in this study can be found in online repositories. The names of the repository/repositories and accession number(s) can be found below: doi: 10.5281/zenodo.14625648, 19D-303D.
