# Supplementary material for: Genomic insights into genetic diversity and seed coat color change in common bean composite populations
Source: Front Plant Sci. 2025 Jan 24;15:1523745. doi: 10.3389/fpls.2024.1523745 (PMC11802580; doi:10.3389/fpls.2024.1523745)
Supplement: Supplementary file 1 [file Table1.docx]

Supplementary Material

Genomic insights into genetic diversity and seed coat color change in common bean composite populations

Eva Plestenjak, Mohamed Neji, Lovro Sinkovič, Vladimir Meglič and Barbara Pipan^*^

*** Correspondence:** Barbara Pipan: [barbara.pipan@kis.si](mailto:barbara.pipan@kis.si)

# Supplementary Data

**Supplementary Table 1.** Total number of reads, mapped reads, mapping rate, Q30, GC content and mean coverage of composite populations and two standard varieties of common bean.

| **CPOP/VAR** | **Sample ID** | **Total reads** | **Mapped reads** | **Mapping rate (%)** | **Q30 Percentage** | **GC Content (%)** | **Mean coverage** |
| --- | --- | --- | --- | --- | --- | --- | --- |
| INCBN_03048 | 94 | 125244359 | 122333285 | 97.7 | 77.0 | 37.0 | 32.3 |
|  | 95 | 115570839 | 113801758 | 98.5 | 76.0 | 37.0 | 30.8 |
| SRGB_00366 | 153 | 115296240 | 111946006 | 97.1 | 73.0 | 36.0 | 29.1 |
|  | 154 | 123407905 | 120313165 | 97.5 | 73.0 | 37.0 | 31.3 |
| SRGB_00189 | 141_A | 112368407 | 109222435 | 97.2 | 80.0 | 36.0 | 29.6 |
|  | 141_B | 106712870 | 104129901 | 97.6 | 80.0 | 36.0 | 28.0 |
|  | 142_A | 107085482 | 104829773 | 97.9 | 80.0 | 36.0 | 28.1 |
|  | 142_B | 111097356 | 109067322 | 98.2 | 81.0 | 36.0 | 29.3 |
| SRGB_00366 | 152_A | 108567708 | 105527125 | 97.2 | 74.0 | 37.0 | 27.2 |
|  | 152_B | 106961624 | 104153100 | 97.4 | 73.0 | 37.0 | 27.4 |
| KIS_Amand | 19D | 114095465 | 111478773 | 97.7 | 79.0 | 37.0 | 30.1 |
|  | 20D_A | 107339499 | 105080688 | 97.9 | 79.0 | 37.0 | 28.2 |
|  | 20D_B | 107426367 | 105010031 | 97.8 | 79.0 | 37.0 | 28.0 |
|  | 21D_A | 27253979 | 26429135 | 97.0 | 80.0 | 37.0 | 6.9 |
|  | 21D_B | 114824688 | 112261687 | 97.8 | 80.0 | 36.0 | 30.3 |
|  | 21D_C | 106593504 | 104143895 | 97.7 | 73.0 | 37.0 | 27.5 |
|  | 22D | 104610004 | 102683320 | 98.2 | 80.0 | 37.0 | 27.8 |
| ETNA | 302D | 101538841 | 100014266 | 98.5 | 80.0 | 36.0 | 26.9 |
| Golden_Gate | 303D | 109524938 | 107288349 | 98.0 | 74.0 | 37.0 | 28.0 |

CPOP, composite population; VAR, standard variety.

**Supplementary Table 2.** The effects of identified SNP on genes as classified by SnpEff program.

| **Impact class** | **Count** | **Percent (%)** |
| --- | --- | --- |
| High | 5365 | 0.03 |
| Low | 184032 | 0.85 |
| Moderate | 192015 | 0.88 |
| Modifier | 21395469 | 98.25 |

**Supplementary Table 3.** Amino acid changes identified by SnpEff software. Rows are reference amino acids and columns are changed amino acids. E.g., Row 'A' column 'E' indicates how many 'A' amino acids have been replaced by 'E' amino acids.

|  | ***** | **?** | **A** | **C** | **D** | **E** | **F** | **G** | **H** | **I** | **K** | **L** | **M** | **N** | **P** | **Q** | **R** | **S** | **T** | **V** | **W** | **Y** |
| --- | --- | --- | --- | --- | --- | --- | --- | --- | --- | --- | --- | --- | --- | --- | --- | --- | --- | --- | --- | --- | --- | --- |
| ***** | **268** |  |  | 17 |  | 30 |  | 14 |  |  | 32 | 37 |  |  |  | 64 | 38 | 39 |  |  | 57 | 34 |
| **?** |  | **13** |  |  |  |  |  |  |  |  |  |  |  |  |  |  |  |  |  |  |  |  |
| **A** |  |  | **12742** |  | 519 | 668 |  | 1360 |  |  |  |  |  |  | 875 |  |  | 1813 | 3742 | 3978 |  |  |
| **C** | 117 |  |  | **2888** |  |  | 473 | 386 |  |  |  |  |  |  |  |  | 620 | 1204 |  |  | 248 | 836 |
| **D** |  |  | 536 |  | **6191** | 3166 |  | 1620 | 1045 |  |  |  |  | 3215 |  |  |  |  |  | 490 |  | 905 |
| **E** | 497 |  | 622 |  | 3321 | **5686** |  | 1583 |  |  | 4037 |  |  |  |  | 1944 |  |  |  | 748 |  |  |
| **F** |  |  |  | 419 |  |  | **4984** |  |  | 733 |  | 3178 |  |  |  |  |  | 1237 |  | 559 |  | 818 |
| **G** | 144 |  | 1404 | 464 | 1830 | 1870 |  | **12698** |  |  |  |  |  |  |  |  | 2071 | 1576 |  | 761 | 149 |  |
| **H** |  |  |  |  | 983 |  |  |  | **3254** |  |  | 577 |  | 1020 | 415 | 1561 | 1129 |  |  |  |  | 1256 |
| **I** |  |  |  |  |  |  | 779 |  |  | **6910** | 302 | 1609 | 1594 | 684 |  |  | 261 | 407 | 2072 | 3385 |  |  |
| **K** | 222 |  |  |  |  | 3014 |  |  |  | 373 | **5964** |  | 513 | 2906 |  | 1332 | 2353 |  | 763 |  |  |  |
| **L** | 255 |  |  |  |  |  | 3875 |  | 578 | 1905 |  | **25069** | 1081 |  | 1330 | 616 | 438 | 1201 |  | 2460 | 229 |  |
| **M** |  |  |  |  |  |  |  |  |  | 2096 | 497 | 1069 |  |  |  |  | 290 |  | 714 | 1026 |  |  |
| **N** |  |  |  |  | 2362 |  |  |  | 871 | 701 | 2635 |  |  | **5395** |  |  |  | 2403 | 810 |  |  | 646 |
| **P** |  |  | 872 |  |  |  |  |  | 457 |  |  | 1875 |  |  | **11011** | 581 | 504 | 2719 | 681 |  |  |  |
| **Q** | 747 |  |  |  |  | 1964 |  |  | 1696 |  | 1545 | 822 |  |  | 557 | **4472** | 1264 |  |  |  |  |  |
| **R** | 301 |  |  | 952 |  |  |  | 1689 | 1264 | 468 | 2575 | 416 | 320 |  | 401 | 1112 | **8952** | 1366 | 662 |  | 441 |  |
| **S** | 404 |  | 1422 | 1316 |  |  | 1664 | 1201 |  | 575 |  | 1408 |  | 2614 | 2183 |  | 1394 | **16832** | 2744 |  | 126 | 878 |
| **T** |  |  | 3087 |  |  |  |  |  |  | 2727 | 792 |  | 602 | 848 | 634 |  | 631 | 2659 | **11127** |  |  |  |
| **V** |  |  | 3100 |  | 427 | 644 | 715 | 644 |  | 4056 |  | 2510 | 1190 |  |  |  |  |  |  | **12066** |  |  |
| **W** | 578 |  |  | 300 |  |  |  | 133 |  |  |  | 328 |  |  |  |  | 416 | 164 |  |  |  |  |
| **Y** | 326 |  |  | 795 | 686 |  | 907 |  | 1123 |  |  |  |  | 649 |  |  |  | 633 |  |  |  | **3629** |

**Supplementary Table 4.** Number of base changes (SNPs), transitions are coloured in red and transversions in green.

|  | **A** | **C** | **G** | **T** |
| --- | --- | --- | --- | --- |
| **A** | 0 | 367272 | 1236799 | 505467 |
| **C** | 432039 | 0 | 272132 | 1524041 |
| **G** | 1525975 | 271734 | 0 | 432796 |
| **T** | 505935 | 1235348 | 370150 | 0 |

**Supplementary Table 5.** The distribution of the total number of ROHs and the distribution per chromosome in the composite populations and the standard varieties of common bean.

| **CPOP/VAR** | **Chromosome** | **Chromosome length (bp)** | **Total ROH length (bp)** | **Number of ROH** | **Mean ROH length (Mb)** | **Coverage (%)** | **ROH (%)** |
| --- | --- | --- | --- | --- | --- | --- | --- |
| ETNA | Chr1 | 52205531 | 32640 | 1575 | 0.021 | 0.040 | 11.99 |
|  | Chr2 | 49040938 | 26861 | 1166 | 0.023 | 0.047 | 8.88 |
|  | Chr3 | 52284309 | 32525 | 786 | 0.041 | 0.079 | 5.98 |
|  | Chr4 | 45960019 | 39378 | 1663 | 0.024 | 0.052 | 12.66 |
|  | Chr5 | 40819286 | 33678 | 936 | 0.036 | 0.088 | 7.13 |
|  | Chr6 | 39171318 | 25469 | 821 | 0.031 | 0.079 | 6.25 |
|  | Chr7 | 53221353 | 28428 | 1229 | 0.023 | 0.043 | 9.36 |
|  | Chr8 | 46493844 | 30182 | 1395 | 0.022 | 0.047 | 10.62 |
|  | Chr9 | 45677737 | 40303 | 1192 | 0.034 | 0.074 | 9.07 |
|  | Chr10 | 55788912 | 40503 | 1154 | 0.035 | 0.063 | 8.79 |
|  | Chr11 | 50079135 | 38619 | 1218 | 0.032 | 0.063 | 9.27 |
| Golden_Gate | Chr1 | 52205531 | 20885 | 1578 | 0.013 | 0.025 | 10.08 |
|  | Chr2 | 49040938 | 17031 | 1229 | 0.014 | 0.028 | 7.85 |
|  | Chr3 | 52284309 | 27152 | 1244 | 0.022 | 0.042 | 7.94 |
|  | Chr4 | 45960019 | 26994 | 1732 | 0.016 | 0.034 | 11.06 |
|  | Chr5 | 40819286 | 23514 | 916 | 0.026 | 0.063 | 5.85 |
|  | Chr6 | 39171318 | 20779 | 1217 | 0.017 | 0.044 | 7.77 |
|  | Chr7 | 53221353 | 19077 | 1350 | 0.014 | 0.027 | 8.62 |
|  | Chr8 | 46493844 | 18655 | 1421 | 0.013 | 0.028 | 9.07 |
|  | Chr9 | 45677737 | 31045 | 1675 | 0.019 | 0.041 | 10.70 |
|  | Chr10 | 55788912 | 31969 | 1750 | 0.018 | 0.033 | 11.17 |
|  | Chr11 | 50079135 | 26669 | 1549 | 0.017 | 0.034 | 9.89 |
| INCBN_03048 | Chr1 | 52205531 | 53526 | 3160 | 0.017 | 0.032 | 10.43 |
|  | Chr2 | 49040938 | 42880 | 2366 | 0.018 | 0.037 | 7.81 |
|  | Chr3 | 52284309 | 57541 | 2331 | 0.025 | 0.047 | 7.69 |
|  | Chr4 | 45960019 | 61839 | 3624 | 0.017 | 0.037 | 11.96 |
|  | Chr5 | 40819286 | 53500 | 2250 | 0.024 | 0.058 | 7.42 |
|  | Chr6 | 39171318 | 44072 | 2267 | 0.019 | 0.050 | 7.48 |
|  | Chr7 | 53221353 | 47307 | 2477 | 0.019 | 0.036 | 8.17 |
|  | Chr8 | 46493844 | 45164 | 3006 | 0.015 | 0.032 | 9.92 |
|  | Chr9 | 45677737 | 70131 | 2716 | 0.026 | 0.057 | 8.96 |
|  | Chr10 | 55788912 | 69219 | 3452 | 0.020 | 0.036 | 11.39 |
|  | Chr11 | 50079135 | 64174 | 2655 | 0.024 | 0.048 | 8.76 |
| KIS_Amand | Chr1 | 52205531 | 208337 | 11087 | 0.019 | 0.036 | 11.57 |
|  | Chr2 | 49040938 | 161155 | 7842 | 0.021 | 0.042 | 8.19 |
|  | Chr3 | 52284309 | 211180 | 6263 | 0.034 | 0.064 | 6.54 |
|  | Chr4 | 45960019 | 256532 | 11505 | 0.022 | 0.049 | 12.01 |
|  | Chr5 | 40819286 | 215699 | 6824 | 0.032 | 0.077 | 7.12 |
|  | Chr6 | 39171318 | 170952 | 6618 | 0.026 | 0.066 | 6.91 |
|  | Chr7 | 53221353 | 185810 | 8893 | 0.021 | 0.039 | 9.28 |
|  | Chr8 | 46493844 | 190597 | 9876 | 0.019 | 0.042 | 10.31 |
|  | Chr9 | 45677737 | 267518 | 9075 | 0.029 | 0.065 | 9.47 |
|  | Chr10 | 55788912 | 254601 | 9012 | 0.028 | 0.051 | 9.41 |
|  | Chr11 | 50079135 | 249065 | 8811 | 0.028 | 0.056 | 9.20 |
| SRGB_00189 | Chr1 | 52205531 | 129195 | 6264 | 0.021 | 0.040 | 11.89 |
|  | Chr2 | 49040938 | 107804 | 4738 | 0.023 | 0.046 | 9.00 |
|  | Chr3 | 52284309 | 129055 | 3100 | 0.042 | 0.080 | 5.89 |
|  | Chr4 | 45960019 | 157730 | 6604 | 0.024 | 0.052 | 12.54 |
|  | Chr5 | 40819286 | 134931 | 3957 | 0.034 | 0.084 | 7.51 |
|  | Chr6 | 39171318 | 102296 | 3406 | 0.030 | 0.077 | 6.47 |
|  | Chr7 | 53221353 | 113932 | 4880 | 0.023 | 0.044 | 9.26 |
|  | Chr8 | 46493844 | 120264 | 5690 | 0.021 | 0.045 | 10.80 |
|  | Chr9 | 45677737 | 160926 | 4670 | 0.034 | 0.075 | 8.87 |
|  | Chr10 | 55788912 | 161369 | 4631 | 0.035 | 0.062 | 8.79 |
|  | Chr11 | 50079135 | 152995 | 4732 | 0.032 | 0.065 | 8.98 |
| SRGB_00366 | Chr1 | 52205531 | 84789 | 6450 | 0.013 | 0.025 | 10.09 |
|  | Chr2 | 49040938 | 68020 | 4982 | 0.014 | 0.028 | 7.79 |
|  | Chr3 | 52284309 | 106237 | 5516 | 0.019 | 0.037 | 8.63 |
|  | Chr4 | 45960019 | 106255 | 6892 | 0.015 | 0.034 | 10.78 |
|  | Chr5 | 40819286 | 92426 | 4171 | 0.022 | 0.054 | 6.52 |
|  | Chr6 | 39171318 | 82016 | 4737 | 0.017 | 0.044 | 7.41 |
|  | Chr7 | 53221353 | 76849 | 5376 | 0.014 | 0.027 | 8.41 |
|  | Chr8 | 46493844 | 75690 | 5536 | 0.014 | 0.029 | 8.66 |
|  | Chr9 | 45677737 | 122710 | 6834 | 0.018 | 0.039 | 10.69 |
|  | Chr10 | 55788912 | 129081 | 7237 | 0.018 | 0.032 | 11.32 |
|  | Chr11 | 50079135 | 106250 | 6199 | 0.017 | 0.034 | 9.70 |

CPOP, composite population; VAR, standard variety.
